# Epidermolysis Bullosa Registry Data in Iran

**DOI:** 10.29252/wjps.10.3.99

**Published:** 2021-09

**Authors:** Siamak Farokhforghani, Mohammad Javad Fatemi, Parinaz Ghanooni, Faraz Asadpour, Shirin Araghi, Afshin Nouri

**Affiliations:** 1 Burn Research Center, Iran University of Medical Sciences, Tehran, Iran.; 2 Hazrat Fatemeh Hospital, Iran University of Medical Sciences, Tehran, Iran.

**Keywords:** Epidermolysis bullosa, Epidemiology, Iran

## Abstract

**BACKGROUND:**

In many countries, there is no registry system to record data for Epidermolysis Bullosa patients. However, the first steps for establishing a registry system have been taken in Iran. Therefore, we decided to publish it for the first time.

**METHODS:**

This was a prospective cross-sectional study. Data was obtained from 538 patients consecutively enrolled in the Iranian Epidermolysis Bullosa Registry, using a detailed instrument created by burn research center of Iran University of Medical Sciences, Tehran, Iran from Jan 2017 to Sep 2017. Patients’ information such as age, gender, address, educational status, parents’ family relationship and pathology result were recorded. Then a physician examined patients focusing on gastrointestinal system, teeth, ophthalmologic disorders, psychological problems and contracture of the upper and lower limbs and any other complaint. Data entered SPSS ver.19 and analyzed using ANOVA and LSD tests.

**RESULTS:**

Overall, 538 EB patients were registered in Iran (6.72 patient in 100.000 person) with an approximately equal ratio between males and females. Among 103 patients whose disease type was determined by a pathologist, 78 patients (75.7%) had dystrophic type, 13 (12.6%) junctional, 9 (8.7%) simplex and 3 (2.9%) kindler type. The most common complaint of patients was dysphagia followed by tooth damage.

**CONCLUSION:**

We stablished a data registry for EB patients for the first time in Iran. The frequency of EB in Iran is less than many other countries. However, data completion is to be done to include all patients as possible.

## INTRODUCTION

Epidermolysis Bullosa (EB) also called “the Butterfly disease” is a group of hereditary bullous disorders characterized by painful skin blisters due to friction or minor trauma. This disease is divided into four primary categories based on membrane and skin affected area including; a) Simple or simplex (detachment is in epidermis); b) Recessive dystrophic (detachment is in lamina lucid layer or basal membranes); c) Recessive junctional (detachment is under lamina dusa basal membrane); d) Hemidesmosomal (detachment is in the most superficial layer of basal membrane in the level of hemidesmosomes)^1^^-^^5^.

 Some infants develop massive blisters at birth while in others blisters appear shortly after birth. Blisters tend to form on weight bearing areas or joints. In mild forms of the disease, it may be diagnosed at adulthood or rarely remained unknown till older ages ^6^^-^^8^.

The simplex type of Epidermolysis Bullosa does not have any extra-dermal involvement or a limited involvement, while hemidesmosomal, junctional and dystrophic types, multiple organs are affected. This uncommon genetic disorder afflicts all races. About 100,000 Americans are living with this disease ^9^^-^^11^. 

According to EB patients’ national registry report, there are 50 cases of this disease in a million live births in America, among which approximately 92% are simplex type, 5% dystrophic, 1% junctional and 2% have no exact category. EB presented statistics is very variable. EB registry system is not available in many countries, also in many countries with such system, all patients are not registered. EB prevalence is estimated as one in 20,000 to one in 100,000 in the Europe and the United States and the incidence is 19 in a million live births ^12^^, ^^13^. 

Patient’s registry system has been recently established in Iran. Therefore, we decided to analyze and report initial statistical data of patients with EB. 

## METHODS

At first, an EB registry system was established in the Burn Research Center of Iran University of Medical Sciences, Tehran, Iran. Then, we started to recall patients using social media and networks from Jan 2017 to Sep 2017. Patients were called and invited to be visited at our center. An informed written consent was obtained from all patients. The study was approved ethically by the university. Patients’ information including age, sex, address, type of disease, EB history in patients’ relatives, and educational degree were recorded. 

Afterwards, the disease type with pathology approval was registered in their file and those without a pathology confirmation were referred to our pathology department for assessment. 

 Then patients were visited by our physicians and examined regarding gastrointestinal system, eyes, teeth diseases, psychological status and limb contracture. Finally, data was analyzed using SPSS ver.19 (IBM software, Chicago. Ill, the USA) by ANOVA and LSD tests. *P*-value less than 0.05 was considered as statistically significant.

## RESULTS

Overall, 538 patients were registered during 9 months. Considering Iran population of 80,000,000 people, EB frequency was found to be 6.72 per 1,000,000. The youngest patient was 10 d old and the oldest one 64 (13.39 11.25) years. In total, 255 (46.5%) patients were female and 288 (52.6%) male. ‌Besides, most patients [173 (31.6%)] were students, 49 (8.9%) had a university education and 34 (6.2%) were illiterate. Overall, the disease type was determined by a pathologist in 103 patients. Among these, the dystrophic type was the most common in 78 patients (75.7%). Other types were junctional in thirteen (12.6%) patients, simplex in 9 (8.7%) and kindler type in 3 (2.9%). Most patients with EB lived in Tehran (47%), Isfahan (8.5%), Khuzestan (7.6%) and Fars (6.7%), respectively (Table 1). 

In dystrophic type group, 39 patients (50%) were male and 39 (50%) female. In simplex EB, 6 (66%) were male and 3 (34%) female and in junctional type, 9 (69%) were male and 4 (31%) female, while all 3 patients with the kindler type (100%) were male. 

 Mean age of patients in the dystrophic group was 12 ± 8.01 years. Mean age in the simplex group was 22 ± 15.07 yr, in junctional type 9 ± 10.03 yr and in kindler type 30 ± 5.00 years. In other patients in which the disease type was not determined, 231 (53%) were male and 209 (47%) female. There were only 4 illiterate EB patients in dystrophic type (5%) and there were no illiterate patients in any other type of EB. Most EB patients were in school age.

Concerning patients’ family relationship, in junctional type, there was a family relationship between parents of all patients. However, this was 84.4% (65 cases) in dystrophic EB, 4 cases (50%) in simplex and 2 cases (67%) in kindler type. ANOVA test showed a meaningful association between patients age and EB type (*P*=0.001).

Mean age in simplex group was 21.12±15.64 yr, which was meaningfully higher than that in both dystrophic (12.27±8.07 yr) and the junctional groups (10.5±10.56 yr) (*P*=0.013 and *P*=0.010, respectively), indicating that simplex patients were older. 

In addition, 270 (80%) patients had different grades of dysphagia, 260 (76%) had teeth diseases, 181 (53%) had some degrees of contracture in the upper and lower limbs and fingers, 160 (47%) had ophthalmological involvement and 48 (14%) psychological disorders. 

## DISCUSSION

Overall, 538 patients were registered during 9 months in our center indicating an EB frequency of 4.2 per 1,000,000 in Iranian population. Besides, the most common pathology type was the dystrophic one. Most patients with EB lived in Tehran. Most patients had different grades of dysphagia. Teeth diseases, contracture in the upper and lower limbs and fingers, ophthalmological involvement and psychological disorders were other common complaints in our patients, respectively. 

The precise prevalence and incidence rate of EB is not known, numerous statistics have been reported from different parts of the world. EB frequency in our research was 4.2 in a million while EB prevalence in Australia is 10 in one million with a mean age of about 14 years. Based on the US national EB registry, the prevalence has been reported to be 8 in one million^14^. It is 9.5 in a million in Croatia^14^ and 8 in a million in Italy^15^. EB prevalence in Europe is about 10 in a million^16^. In Scotland, a higher prevalence has been reported compared to other areas^17^, which might be due to more precise patient screening. This rate was lower in our country, which might be due to under-diagnosis, since our patients were registered only during nine months. Usually, some patients may not have been referred to our EB center leading to an underestimation^14^. 

As it was expected, the number of male and female patients were approximately the same. About 60% of our patients lived in 6 big provinces such as Tehran, Alborz, Khorasan Razavi, Isfahan, Fars and Khouzestan and about 40% were habitants of small provinces. This was approximately consistent with Australia statistics indicating that 69% of patients lived in big cities and 31% in small ones^3^. Dystrophic type of EB included 78% of patients which was much higher in comparison with India (17%)^18^, Canada (35%)^19^ and Australia (35%)^20^. 

The frequency of junctional type in our study was about 11%; this was 11% in Canada^19^ and Australia^20^, which is consistent with our statistics. The kindler type frequency was about 3% in our study, which was 0.3% in Australia^20^. To our knowledge, the frequency of junctional and kindler types did not differ significantly from other countries, but it was different in dystrophic and simplex types^14^. According to previous studies, the most common type was reported to be the simplex one. 

In Iran, based on our results, the dystrophic type was two-times more prevalent than countries such as Canada and Australia. Besides, the simplex type was much less common than other countries. This might be due to a diagnosis overlap between different EB types. However, some patients with the simplex type might have been misdiagnosed as dystrophic type, because the immunofluorescence test was not performed and electronic microscope was not used. Moreover, patients with dystrophic type tend to refer to health centers more probably as the disease is more severe than the simplex type. Nonetheless, exact diagnostic tests should be performed in all patients to determine the disease type more precisely. 

**Table 1:** Frequency of Epidermolysis Bullosa in Different Provinces in 2017 in Iran

Location/Province	Frequency	Percent
Tehran	109	12.1
Alborz	29	5.3
Qom	16	2.9
East Azerbaijan	22	4.1
Khorasan	43	7.9
Hamedan	30	5.5
Mazandaran	14	2.6
Lorestan	23	4.2
Semnan	6	1.1
Markazi	7	1.3
Isfahan	44	8.1
Fars	23	6.1
Boushehr	6	1.1
Gilan	10	1.8
Ardebil	5	0.9
Kermanshah	4	0.7
Ilam	4	0.7
Charmahal-bakhtiari	6	1.1
West azerbaijan	12	2.2
Khoozestan	43	7.9
Kordestan	8	1.5
Zanjan	13	2.4
Sisatan-balouchestan	15	2.8
Kerman	23	4.2
Qazvin	7	1.3
Yazd	6	1.1
Golestan	2	0.4
Hormozgan	1	0.2
Zahedan	2	0.4
Missing	5	0.9
Total	543	100.00

## CONCLUSION

According to our registry system, Epidermolysis bullosa prevalence in Iran is less than other countries, probably increased with better screening. Most patients lived in 4 provinces of Tehran, Isfahan, Khouzestan and Fars, therefore it is suggested to provide facilities for these patients in these four provinces. Also, dystrophic type of EB was more common in Iran than other countries. For this reason, disease prevention by genetic analysis and genetic tests during pregnancy is of significant importance. Precise diagnosis of the disease type by immunofluorescence tests and electron microscope would help to determine the exact prevalence of disease types. The number of simplex type would be gradually increased by a complete registry of almost all patients. 

## CONFLICT OF INTEREST

 The authors declare no conflict of interest.
